# Ex vivo resection and temporary portocaval shunt of unresectable hepatocellular carcinoma followed by autotransplantation of liver: a case report

**DOI:** 10.1186/s12957-019-1781-7

**Published:** 2020-01-06

**Authors:** Hesameddin Eghlimi, Peyman Arasteh, Alireza Shamsaeefar, Hamed Nikopour, Sahar Sohrabi, Saman Nikeghbalian

**Affiliations:** Shiraz Transplant Research Center, Shiraz University of Medical Sciences, Shiraz, Iran

**Keywords:** Ex vivo, Resection, Hepatocellular carcinoma, Autotransplantation

## Abstract

**Background:**

Ex situ liver resection and autotransplantation is among the most advanced techniques which has been introduced in recent years.

**Case presentation:**

A 24-year-old male referred with chief complaints of abdominal pain, nausea, and vomiting from 1 month prior to admission. Computed tomography showed a large liver mass in the left lobe of the liver with involvement of retrohepatic inferior vena cava (IVC), in favor of hepatocellular carcinoma.

After hepatectomy, the common bile duct was completely removed. A 4-cm Dacron graft was anastomosed to the inferior and top of the IVC. A temporary portocaval shunt was placed, and ex situ resection of the left lobe of the liver was done. Remnant of the liver was implanted. Reconstruction of the bile duct was done using a Roux-en-Y technique, and autotransplantation of the liver was then completed. During a 4-year follow-up, the patient had no complaints and is in good conditions.

**Conclusion:**

With appropriate consideration of patients, despite surgical complexities, ex situ resection of unresectable HCC can provide excellent prognosis.

## Background

Hepatocellular carcinoma (HCC) treatment is widely based on the stage of the cancer. Although surgical resection is considered the optimal treatment, only a few patients qualify for surgery and this is associated with high rates of recurrence and poor intraoperative exposure [1].

Ex situ liver resection is considered a solution to overcome these difficulties. This type of surgery was first performed by Rudolf Pichlmayr in 1988 [2]. To this approach, three techniques are recognized: (1) “in situ” resection and hypothermic perfusion of liver and cross-clamping without any dissection of major vessels, (2) the “ante situm” resection that includes the isolated dissection of the suprahepatic inferior vena cava, (3) and finally the “ex situ” resection that includes a complete removal of the liver and resection as a bench procedure. The third technique has a variation. In this technique, first, the liver is resected, after which ex situ surgery is performed on the part of the liver which includes the tumor. Finally the part of the liver segments which do not include the tumor are re-transplanted [3, 4].

Inhere we report a case of ex vivo resection of HCC and autotransplantation who showed excellent postoperative results.

## Case presentation

A 24-year-old male referred with chief complaints of abdominal pain, nausea and vomiting, and a sensation of fullness from 1 month days prior to his admission.

The patient did not report any diseases or hospitalizations in his past medical history; furthermore, the patients did not report any diseases in his family history.

On physical examination, no abnormal finding was detected. The patient only expressed mild epigastric pain during examination.

Lab data showed a normal liver function test as aspartate aminotransferase (AST) of 33, alanine aminotransferase (ALT) of 26, alkaline phosphatase (ALP) of 265, total serum bilirubin of 1.7, and direct bilirubin of 0.9. Alpha fetoprotein (AFP) was 720.

**Fig. 1:**
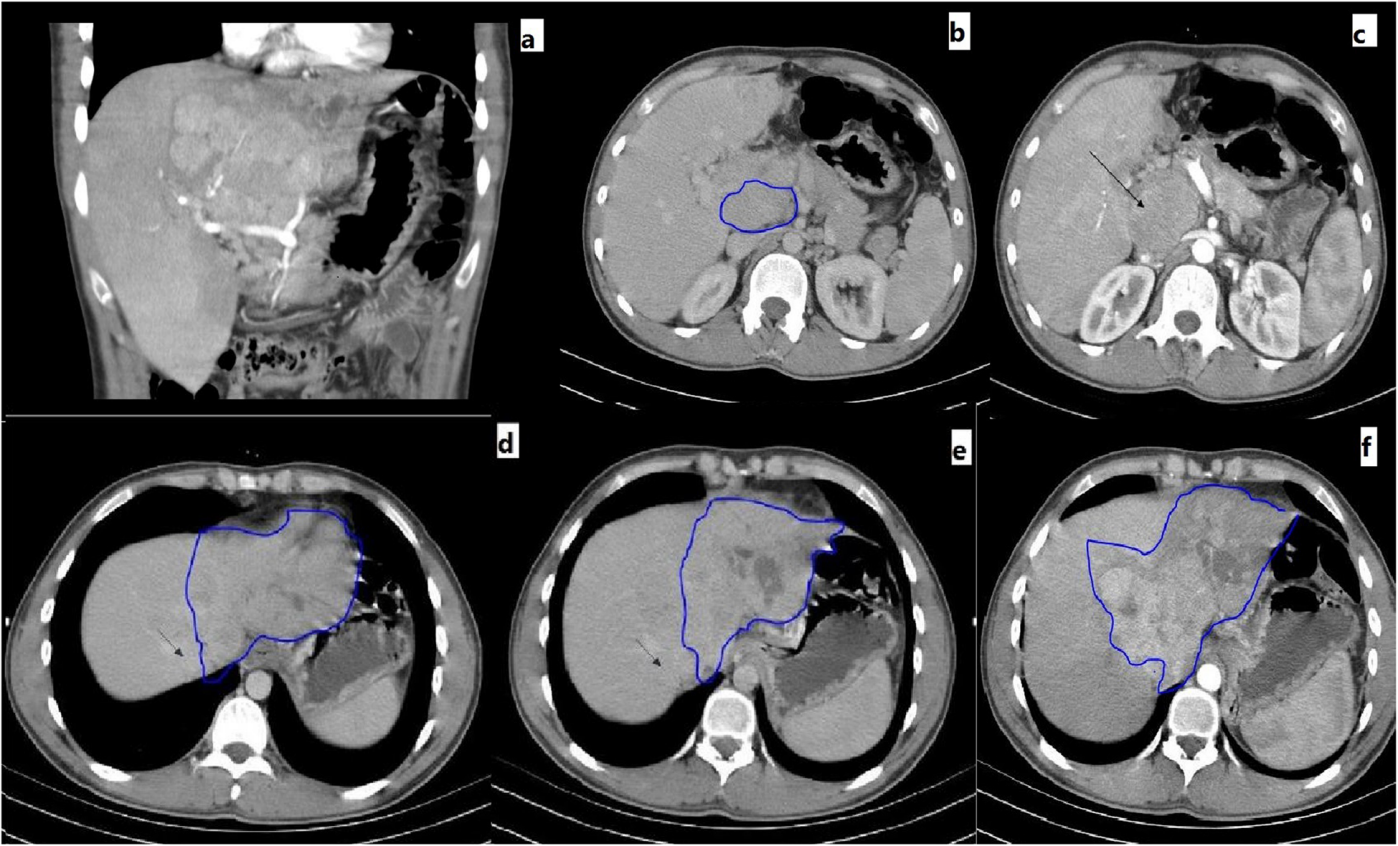
CT scan images of the involved liver. **a** HCC involving the liver from a coronal view. **b**, **c** Multiple lymph nodes in the hilum of the liver (marker shows the largest lymph node). **d**, **e** Tumor involvement of the left and middle hepatic veins and neighboring of the right hepatic vein (arrow). **f** HCC of the left lobe of the liver with involvement of retrohepatic IVC and caudate lobe

During work-up, abdominal sonography and computed tomography (CT) was done which showed a large liver mass in the left lobe of the liver (segments: 2, 3, 4a, 4b, caudate lobe with involvement of retro hepatic inferior vena cava) which was in favor of HCC (Fig. 1).

Chest CT scan and bone scan was done for assessment of distant metastasis which was negative for metastasis.

The patient underwent laparotomy and exploration was done which showed a large mass (20 cm) in the left lobe of the liver and in the caudate lobe with involvement of IVC and involvement of left and middle hepatic veins.

Hepatectomy was done using a standard technique with extensive lymph node dissection around the superior mesenteric artery and celiac trunk. The common bile duct was completely removed up to the head of the pancreas. Frozen sections were sent from distal of the common bile duct which was negative for malignancy. After hepatectomy, a 4-cm Dacron graft was anastomosed to the inferior and top of the IVC so continuity of the IVC would be maintained. For prevention of bowel edema and a hepatic phase, a temporary portocaval shunt was placed, and ex situ resection of the left lobe of the liver was done with the Cavitron Ultrasonic Surgical Aspirator (CUSA) by a bipolar manner. Extension of the portal vein was done using a cadaver vessel bank.

Remnant of the liver (right lobe) was implanted similar to a liver transplant. The right hepatic vein, portal vein, and hepatic artery were anastomosed. Reconstruction of the bile duct was done using the Roux-en-Y technique, and autotransplantation of the liver was then completed.

Hemostasis was achieved and two JP drains were inserted. After which, closure of the abdomen was done and the patient was then transferred to the surgical intensive care unit. The cold ischemic time of the operation was about 90 min, and the warm ischemic time was 45 min.

Pathology reported fibrolamellar HCC with well differentiation, tumor measuring 12 × 11 × 10 cm and 5 × 5 × 4 cm, with multifocal lymphovascular and perineural invasion. The surgical resected margin was free.

**Fig. 2:**
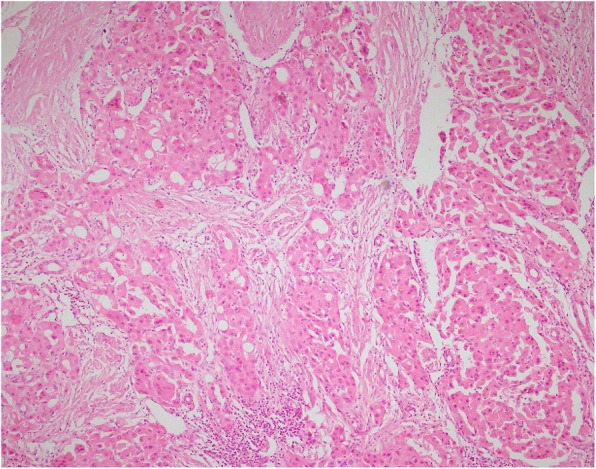
Microscopic section from hepatocellular carcinoma shows tumoral cells arranged in glandular and acinar pattern (hematoxylin and eosin, × 200)

Moreover, pathology also reported a mass in the hilum of the liver measuring 5 × 5 × 4 cm which includes a white appearance with hemorrhage and necrosis. Multiple lymph nodes ranging from 0.5 to 2 cm. None of the 31 dissected lymph nodes were involved with the tumor. Figure 2 shows a microscopic section from the HCC.

Other than intraoperative bleeding, the patient did not develop any major complications during the operation and the postoperative period. The patient was discharged after 14 days of hospital admission with a good overall condition and was referred to the surgery clinic for postoperative visits. Moreover, the patient was given adjuvant chemotherapy for a total of 6 weeks.

During a 2.5 year follow-up the patient showed lymph nodes in the hilum of the liver in imaging, for which he underwent laparotomy, and three lymph nodes were removed. In pathology evaluation, one lymph node was involved with the tumor.

**Fig. 3:**
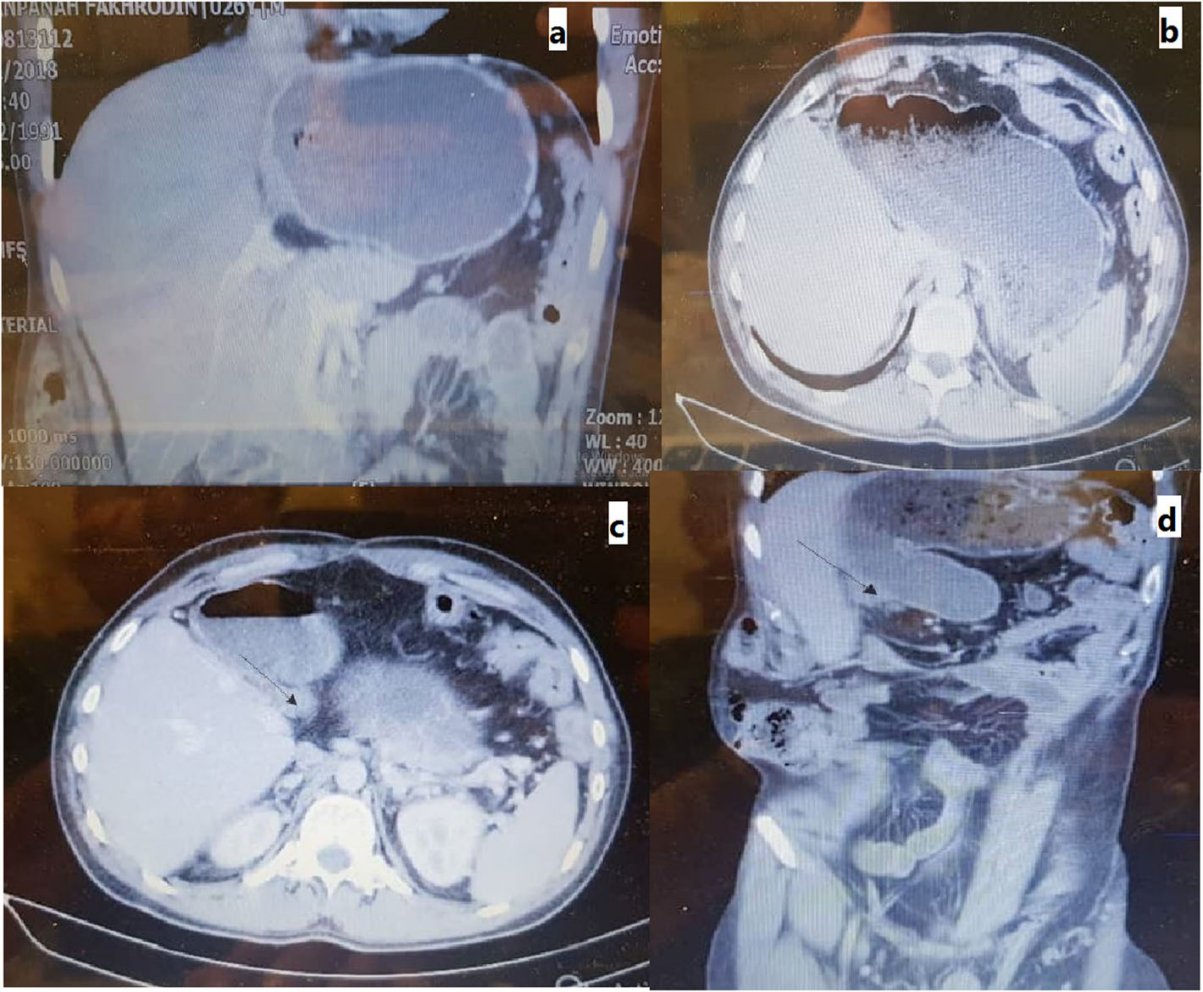
Postoperative Ct scan images of patient at 2.5-year follow-up. **a**, **b** The liver with no lesions and no tumor involvement. **c**, **d** Arrows show one large lymph node in the hilum of the liver with possibility of recurrence or remnant of previously involved lymph node

During a 4-year follow-up, no recurrence of primary tumor was recorded, and the patient had no complaint and was in good conditions. Figure 3 shows postoperative CT scan during follow-up.

Additional files 1 and 2 show gross description of the tumor and surgery specifics.

## Discussion and conclusion

During recent years with advances in surgical techniques, liver function after surgical resection of tumors has improved significantly. The liver has a specific limitation for ischemic tolerance, and when the tumor is difficult to access, in cases of proximity to venous confluences or main hepatic veins, the use of conventional surgical techniques becomes problematic [3].

Although multiple studies have reported on ex situ resection and autotransplantation [5], very few studies have evaluated the results of ex situ resection among patients with HCC [6–8], among which Wen at el. had the largest report including three patients with HCC who underwent this specific procedure [8]. Our patient had extensive involvement of the liver which included the IVC as well, and compared to previous literature, we had the longest follow-up (4 years) through which the patient reported no complaints or complications.

This technique is considered to be associated with a high rate of mortality with a median survival of 25 months [5] and overall 90-day mortality rate of 19.5% [9]. Mortality is mainly attributed to factors like liver failure and sepsis [5]. The most common complications after ex situ liver resection other than sepsis and liver failure include bleeding and biliary leakage [10]. Among patients who have the procedure for tumors, during long term, the biggest issue remains to be recurrence of the primary tumor [11].

Preoperative evaluation of patients who are candidates for this surgical technique is important, as this technique should only be considered for patients who have a normal liver function with non-resectable tumors [4].

With appropriate consideration of patients, despite surgical complexities, ex situ resection of unresectable HCC can provide excellent prognosis.

## Supplementary information

**Additional file 1.** This video shows the following sequences consecutively: Gross description of liver anatomy and HCC involvement; Hepatic vein involvement by HCC; Inferior vena cava involvement by HCC; Closure of orifice of middle hepatic vein.

**Additional file 2.** This video shows the following sequences consecutively: Partial clamping of the IVC and prepping for anastomosis with portal vein; anastomosis of IVC and portal vein; transection phase at liver bench phase; right lobe of liver after tumor resection; and a phase after liver implantation and after anastomosis of port and hepatic artery.

## Data Availability

N/A.

## Abbreviations

ALP: Alkaline phosphatase
ALT: Alanine aminotransferase of 26
AST: Aspartate aminotransferase
HCC: Hepatocellular carcinoma
